# Functional Properties and Preservative Effect of P-Hydroxybenzoic Acid Grafted Chitosan Films on Fresh-Cut Jackfruit

**DOI:** 10.3390/foods11091360

**Published:** 2022-05-07

**Authors:** Zhiguo Jiang, Jiaolong Wang, Dong Xiang, Zhengke Zhang

**Affiliations:** 1 College of Food Science and Engineering, Hainan University, Haikou 570228, China; jzg6666@163.com (Z.J.); jzg6666@sina.com (J.W.); 2 Key Laboratory of Food Nutrition and Functional Food of Hainan Province, Hainan University, Haikou 570228, China

**Keywords:** conjugated chitosan film, functionality, preservation effect

## Abstract

In the present study, p-hydroxybenzoic acid-grafted chitosan (PA-g-CS) conjugates with different grafting degrees were synthesized by a free radical-regulated grafting approach. The conjugates were further developed into films by casting, and their characteristics and preservative effects on fresh-cut jackfruit were evaluated. Compared to the CS film, the PA-g-CS film showed comprehensive performance improvements, including enhancements of water solubility, anti-ultraviolet capacity, antioxidation, and antibacterial activity. Moreover, compared with CS film, some appreciable and favorable changes of physical properties were observed in the PA-g-CS films, which included water vapor permeability, oxygen permeability, surface morphology, moisture content, and mechanical intensity. Furthermore, compared to CS alone, the application of PA-g-CS films to fresh-cut jackfruit exerted a beneficial effect on the quality of products, as indicated by the inhibition of weight loss, softening, and membrane damage, the maintenance of soluble solids and ascorbic acids contents, as well as a reduced bacterial count and a higher sensory score. Among these PA-g-CS films, the best preservation effect was achieved with the highest degree of grafting (PA-g-CS III). The results suggested that the PA-g-CS film has the potential to be explored as a new type of packaging material for the preservation of fresh-cut fruits and vegetables.

## 1. Introduction

Jackfruit (*Artocarpus heterophyllus* L.) is one of the world’s largest fruits, and each mature tree may produce about 10 to 200 individual fruits [1]. Nevertheless, the non-edible part accounts for approximately 60% of the whole fruit, which is regarded as unavailable waste comprised of a prickly rind, an inner perianth, and the central core [2]. Peeling jackfruit is time-consuming and laborious for consumers. Ready-to-eat freshly cut fruits and vegetables have been well accepted by customers on a global scale, owing to advantages including convenience, health, diverse flavor, and nutrients from the collocation of different fruits, and the reduction of waste [3]. On the downsides, fresh-cut fruits deteriorate faster than unprocessed raw fruits, which is primarily attributed to the damage arising from the processing operations such as peeling, slicing, shredding, and dicing. Usually, the shelf life of fresh-cut vegetables and fruits is shortened, during which typical deterioration symptoms are shown by browning of the cut surface, accelerated tissue softening, decreased flavor, and reduced nutritional value [4,5,6,7]. Besides, the exposure of the cut surface to air facilitates certain microbial pathogen development in the fresh-cut products, which can easily cause food-borne diseases. Due to the short shelf life of fresh-cut jackfruit flesh, the industrial production of the fruit is greatly restricted. Hence, the application of adequate preservative measures is being sought for achieving a prolonged post-harvest storage period, along with the quality conservation of the fresh-cut flesh of the jackfruit.

Cationic polysaccharide chitosan (CS) is obtained by the deacetylation of chitin, and it is considered an excellent biomaterial for edible coatings. Manufacturing food packaging film is beneficial and practical due to its nontoxicity, film-forming ability, biocompatibility, biodegradability, antimicrobial activity, and antioxidative activity [8,9,10]. However, the limited antioxidant and antimicrobial activities of CS film alone cannot satisfy effective fresh-cut jackfruit preservation, because they exert poor protection for food from oxidation and microbial infection. This necessitates the addition of antimicrobial and antioxidant agents in the CS films to upgrade their physical properties and functional performance, widening their application in food packaging [11]. Since CS has amino and hydroxyl reactive functional groups, it is feasible to incorporate new features into it through chemical modification techniques [12]. The most useful and prominent technique to modify the intrinsic properties of polysaccharides, particularly natural polymers, is recognized as graft copolymerization [13]. With this technique, the intrinsic properties of CS can be improved while imparting a few new features, such as mucoadhesivity, anti-ultraviolet capacity, and mechanical intensity [14,15,16]. Many researchers have studied the grafting of phenolic acids to CS and its derivatives [17]. The antioxidant activity of CS can be remarkably enhanced by the covalent binding of phenolics [18]. Furthermore, these fabricated phenolic-grafted CSs can be processed into antioxidant films for food packaging. According to the extant reports, the major factors influencing the antioxidant performance and physical properties of the phenolic-grafted CS films include the grafting ratio and the type of bound phenolics [19,20,21,22].

P-hydroxybenzoic acid (PA), a monohydroxy phenolic derivative of benzoic acid, is widely used as an antioxidant, preservative, and fungicide in foods, beverages, medicines, and cosmetics [23,24]. Our previous studies have shown that inserting p-hydroxybenzoic acid (PA) into the CS molecule can enhance its antioxidation and antibacterial abilities [25].

However, to our knowledge, no study has investigated the impact of PA-g-CS based film on the preservation of fresh-cut jackfruit during storage. In this study, a free radical-mediated method was used to graft PA onto CS, which was accomplished using an ascorbic acid (Vc)-hydrogen peroxide (H_2_O_2_) redox system in an inert atmosphere. Then, the PA-g-CSs with varying grafting ratios were cast into the films. Later on, the physical properties (moisture content, and water solubility), mechanical behaviors (elongation at break and tensile strength), barrier performance (water vapor permeability, oxygen permeability, and optical transmittance), biological activities (antioxidation, and antibacterial activity), and surface morphologies of the prepared PA-g-CS films were evaluated for the first time. Finally, we applied the films onto the fresh-cut jackfruit and evaluated the preservative effects of chitosan and PA-g-CS films.

## 2. Materials and Methods

### 2.1. Materials and Reagents

Jackfruits at 80% maturity (light bluish green) were picked from a commercial farm in Haikou, China, and the fruits with uniform sizes were selected for the study. Chitosan with a 95% deacetylation degree and a dynamic viscosity coefficient of 100 to 200 mPa was provided by Macklin Biochemical Co., Ltd. (Shanghai, China). Folin–Ciocalteu reagent (98% purity), p-hydroxybenzoic acid (95% purity), ascorbic acid (99% purity), and 30% H_2_O_2_ (99% purity) were provided by Aladdin Bio-Chem Technology Co., Ltd. (Shanghai, China). All remaining reagents were analytically pure. 

### 2.2. Preparation of PA-Grafted Chitosan

The preparation of PA-g-CS was carried out according to our previous method [25]. Here, 1.2 g of CS was dispensed into a 3-necked round-bottom flask (250 mL) filled with 100 mL of acetic acid aqueous solution (2%, *v*/*v*). Later, varying amounts of PA were added to the above mixture, resulting in different CS-to-PA molar ratios of 1:0.3, 1:0.7, and 1:1. Simultaneously, 0.2 g of ascorbic acid (Vc) was added to all the reactors. The reactor was then injected with oxygen-free nitrogen gas for 30 min under stirring conditions, followed by the addition of 1 mL of 30% H_2_O_2_ solution. This was incubated for 12 h in an oxygen-free nitrogen atmosphere. Later, distilled water was used to dialyze the above reaction mixture for 72 h using a membrane (threshold molecular weight = 8000–14,000 Da) to eliminate the unreacted phenolic acids and additional soluble substances. Finally, PA-g-CS was obtained by lyophilizing the dialysate with different ratios, which were referred to as PA-g-CS-I, PA-g-CS-II, and PA-g-CS-III, respectively. The cold trap temperature of the freeze dryer (Sublimator 15, Bad Grund, Germany) was −30~−40 °C, the sublimation temperature was 50~60 °C, and the time was 8–10 h. The content of polyphenols in the product was detected using the Folin–Ciocalteu method. The degree of grafting was determined to be 35.16 ± 2.07, 65.02 ± 2.34, and 101.16 ± 4.46 (mg PA/g PA-g-CS), respectively.

### 2.3. PA-g-CS Film Preparation

First, PA-g-CS (2% *w*/*v*) was dissolved in 150 mL of acetic acid aqueous solution (1% *v*/*v*). Then, this solution was mixed with 50% (on a dry-weight basis of PA-g-CS) glycerol plasticizer and stirred for 60 min at 25 °C. The resulting mixture was ultrasonicated for 30 min at 200 W and 40 kHz for air bubble elimination. Later, the solution was poured into a 24 cm × 24 cm size glass plate and allowed to dry for 48 h at 40 °C. Finally, the dried PA-g-CS films were removed from the plate and stored at the relative humidity (RH) of 50 ± 2% and a temperature of 25 °C for a minimum of 48 h. Similarly, the control CS films were also prepared.

### 2.4. Characterization of PA-g-CS Films

#### 2.4.1. Fourier Transform Infrared Spectroscopy (FTIR)Analysis

Blank CS, as well as PA-g-CS, films was subjected to dispersion within KBr, pelletization, and analysis with a Bruker Tensor 27 infrared spectrometer (Bruker Inc., Germany) in the range of 4000–400 cm^−1^. 

#### 2.4.2. Scanning Electron Microscopy (SEM) of Films

Once the film surfaces were fractured with liquid nitrogen soaking, they were coated with a thin layer of gold. A S-4800 SEM system (Hitachi, Japan) was utilized to examine the surface morphologies of the film samples at an accelerating voltage of 10 kV.

#### 2.4.3. Light Transmittance

Each rectangular film sample (1 cm × 4 cm) was attached tightly to the inner wall of the cuvette for the measurement of optical transmittance with a double beam spectrophotometer (Shimadzu, UV-2450) at 200–800 nm. An empty cuvette was used as the control. Film opacity was calculated based on the 600 nm absorbance by the following formula [26]:

$$\mathrm{Opacity}\;=\frac{A}{X}$$

where *A* represented absorbance of the film at 600 nm, while *X* represented the thickness of the film (mm). Accordingly, the opacity results were presented as A_600_/mm.

#### 2.4.4. Moisture Content (MC)

In this study, MC was referred to as the weight loss percent of the film after 24 h of drying at 105 °C. The computational formula used to calculate the MC value was as follows [27]:

$$\mathrm{MC}\left(\%\right)=\frac{W_{0}-W_{1}}{W_{0}}\times 100$$

where *W*_0_ (g) was the initial equilibrium weight of the film (2 cm × 2 cm), while *W*_1_ (g) was the weight after the film was dried at 105 °C for 24 h.

#### 2.4.5. Water Solubility (WS) 

The percentage of the dissolved substance following 24 h of thorough film immersion in distilled water was referred to as WS. The computational formula used to calculate the WS value was as follows:

$$\mathrm{WS}\left(\%\right)=\frac{W_{0}-W_{1}}{W_{0}}$$

where *W*_0_ (g) was the initial equilibrium weight of the film (2 cm × 2 cm), while *W*_1_ (g) was the undissolved substance weight following 24 h of film immersion.

#### 2.4.6. Water Vapor Permeability (WVP)

To measure the WVP of films, Liu et al.’s [19] method was employed, with slight modifications. After the sectioning, each film sample was sealed in a glass cup containing thoroughly dried silica gel. The cup was arranged in a 20 °C desiccator filled with distilled water, followed by three consecutive days of weighing at 4 h intervals to obtain a temporal graph of the weight gain. The computational formula to calculate the WVP was as follows:

$$\mathrm{WVP}\;=\frac{C\times x}{A\times \Delta P}$$

where *C* represented the slope of the temporal graph of weight gain (g/s), *x* denoted the thickness of the film (m), *A* denoted the permeation area (m^2^), while Δ*P* was the difference between the partial vapor pressure of pure water and dry atmosphere with a value of 2339 Pa at 20 °C. The WVP results were presented as g/(m·s·Pa).

#### 2.4.7. Oxygen Permeability (OP)

Determination of the OP was accomplished as per the procedure found in the literature [28]. Every 3 g of deoxidizer, comprised of reduced iron powder, sodium chloride, and activated carbon (0.5:1.5:1), was placed onto the bottom of a weighing bottle, and then the bottle was capped with the film and sealed using parafilm. After recording the initial weight of the bottle, all weighing bottles were arranged into a 25 °C desiccator, whose bottom contained 90% RH saturated solution of barium chloride, for 48 h. The computational formula to calculate the *OP* for the film was as follows:

$$OP=\frac{m_{1}-m_{0}}{t*A}$$

where *m*_1_ represented the final weight of the bottle following 48 h of equilibration, *m*_0_ represented the initial bottle weight, *t* denoted the equilibration duration(s), and *A* denoted the exposed surface area of the film with a value of 0.00049 m^2^.

#### 2.4.8. Mechanical Properties of the Film

A tensile tester (Instron 3345, Canton, MA, USA) was used to measure the elongation at break (*EAB*) and tensile strength (*TS*) of the films [19]. Before the tensile experimentation, each film sample was sectioned into 3 rectangular bars with a size of 10 mm × 40 mm. A 0.001 mm precision micrometer was used to record the thickness of each rectangular film. During the tensile experimentation, the initial grip separation was set to 20 mm, while the traction speed was set at 5 mm/min.

#### 2.4.9. DPPH Radical Scavenging Activity

A DPPH assay was employed to determine the antioxidation activity of the film samples [19]. Varying amounts of each film sample were incorporated into a 100 µM DPPH methanol solution (4.0 mL). The equivalents (1.0, 2.0, 3.0, 4.0, and 5.0 mg/mL) of each film sample were added into the reaction liquid. The room-temperature reaction was implemented for 60 min in the dark, followed by the determination of the reaction mixture at an absorbance of 517 nm. The computational formula used to calculate the DPPH radical scavenging activity was as follows:

$$\mathrm{DPPH}\;\mathrm{radical}\;\mathrm{scavenging}\;\mathrm{activity}\;\left(\%\right)=\frac{A_{0}-A_{1}}{A_{0}}\times 100$$

where *A*_0_ and *A*_1_ represented the absorbance values of the control and the supernatant in the sample tube, respectively.

#### 2.4.10. Antibacterial Activity

To determine the antibacterial activity of the film samples, the agar diffusion technique was employed [29]. Here, 100 uL of 10^6^ CFU/mL *E. coli* or *S. aureus* suspensions were spread onto Mueller–Hinton agar. Before the preparation of the film samples, all instruments, including the puncher, were ethanol-sterilized (75%) and UV-irradiated for 30 min in a laminar flow cabinet. The puncher was used to prepare an 8 mm diameter film of circular shapes, which were then subjected to 30 min of UV irradiation for disinfection. These films were then transferred onto the sterilized Petri dishes. Subsequently, the film samples were placed onto the inoculated agar plates in the laminar flow cabinet using sterilized forceps. The samples were then allowed to stand at room temperature for 30 min to diffuse the bioactive compounds from the films to the medium. The plates were incubated for 24 h at 37 °C. Finally, the clear zone diameter at the periphery of the film was measured and recorded in mm, including the diameter of the disk.

### 2.5. The Effect of CS and PA-g-CS Film on the Quality of Fresh-Cut Jackfruit

#### 2.5.1. Jackfruit Preservation Treatment

The jackfruit bud was taken out using a sharp stainless steel knife and immersed in a solution of sodium hypochlorite (100 mg/L) for 30 s. The buds with a uniform shape and size were selected for packaging after being dried for 2 h at room temperature. These jackfruit buds were randomized into five groups: (1) control without packaging treatment; (2) PA-g-CS-I film packaging; (3) PA-g-CS-II film packaging; (4) PA-g-CS-III film packaging; (5) CS film packaging. The jackfruit flesh was tightly wrapped with the respective films and then placed in a 17.5 cm × 12 cm × 7 cm plastic basket. All the groups were then stored at a temperature of 15 °C with a relative humidity (RH) of 80–90%. 

#### 2.5.2. Measurement of the Weight Loss in the Fruit

To determine the net weight, an analytical balance was utilized to weigh the entire individual package of jackfruit bulbs on the day of the observation. The values were presented as the percentage of weight loss. 

#### 2.5.3. Hardness of the Fruit

The measurement of hardness of the flesh was accomplished as per Zheng et al.’s approach, with slight modifications [30]. The maximum force of the force-time graph was determined using a TA.XT2i texture analyzer (Stable Micro Systems, Guildford, UK) with a P/2 probe (diameter 2 mm). The jackfruit flesh was penetrated at the speed of 1 mm/s to a depth of 5 mm.

#### 2.5.4. Total Soluble Solids (TSS)

The jackfruit pulp was homogenized in distilled water at a ratio of 1:4. The homogenized liquid was centrifuged at a speed of 5000 rpm/min for 15 min. The TSS of the supernatant was measured with a handheld Brix meter.

#### 2.5.5. Estimation of Ascorbic Acid

A total of 10 g of the sample was extracted with 3% metaphosphoric acid and the volume was supplemented up to 100 mL. Subsequently, 10 µL of the filtrate was titrated with 2.6-dichlorophenolindophenol dye (0.025%) until the distinct rose pink color persisted for 15–20 s. The results were expressed as the average value of three replicate samples. 

#### 2.5.6. Measurement of the Total Bacterial Count (TBC)

The colony counting technique was employed to examine the TBC of the jackfruit, as per the GB 4789.2–2016 of the Chinese standard. The result was expressed as log CFU/g.

#### 2.5.7. Membrane Permeability

The membrane permeability was presented in terms of relative conductivity and measured as described by Chen et al., with slight modifications [31]. First, pulps with a size of 10 mm diameter (8 pieces, about 2 g per piece) were removed from the flesh by a punch. The pulps were rinsed with distilled water three times and then wiped with filter paper. Next, the initial conductivity (C_1_) was determined after the pulps were placed in a beaker containing 20 mL of distilled water for 30 min. Finally, the conductivity (C_2_) was measured after 15 min of boiling and subsequent cooling at room temperature. The computational formula to calculate the electrolyte leakage rate was as follows:

$$\mathrm{Leakage}\;\mathrm{rate}\;\left(\%\right)=\frac{C_{1}}{C_{2}}\times 100$$

#### 2.5.8. Sensory Evaluation

The procedure for sensory evaluation was taken from Hu et al. [32]. Panelists (3 males and 3 females) were requested to score the organoleptic properties such as color, odor, appearance, texture, and overall acceptability on a ten-point scale (10–9 points: excellent, 8–7 points: good, 6–5 points: acceptable, 4–3 points: poor, and 2–1 points: very poor).

### 2.6. Statistical Analysis

All the experiments were performed in triplicate. The values were expressed as means ± standard deviation. Data were analyzed using SPSS 25. The significance of the inter-sample comparisons was determined using Duncan’s multiple range test and the analysis of variance (ANOVA); *p*-values less than 0.05 were regarded as statistically significant.

## 3. Results

### 3.1. The Properties of Films

#### 3.1.1. FT-IR Spectra

**Figure 1:**
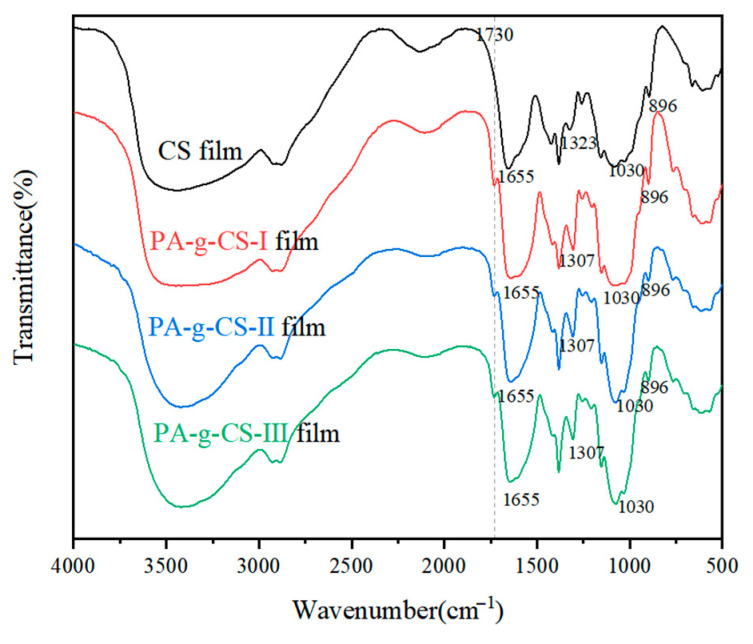
FT˗IR spectra of CS and PA-g-CS films.

Figure 1 presented the FT-IR spectra for CS and PA-g-CS films. All curves displayed representative bands of amino polysaccharides. The CS film showed characteristic bands around 3450 cm^−1^ (OH- and NH- stretching in the benzene ring), 2930–2870 cm^−1^ (C-H stretching), 1655 cm^−1^ (amide-I, C=O stretching), 1590 cm^−1^ (amide-II, N-H bending), 1323 cm^−1^ (amide-III, C-N stretching, residual N-acetyl groups), 1030 cm^−1^ (C-O-C), and 896 cm^−1^ (pyranose ring). This finding was consistent with previous reports in the literature [33]. The three conjugates showed a novel band at 1730 cm^−1^, which was related to C=O group stretching of the saturated ester. This observation indicated the occurrence of graft co-polymerization of PA (-COOH) and CS (-OH), along with the formation of ester linkages. Moreover, it was observed that the bands of the three conjugates in amide-III shifted from 1323 to 1307, with higher peak intensity than that of the CS film. This also confirmed that the grafting reaction occurred in the amino group (-NH_2_). 

#### 3.1.2. Film Microstructure

**Figure 2:**
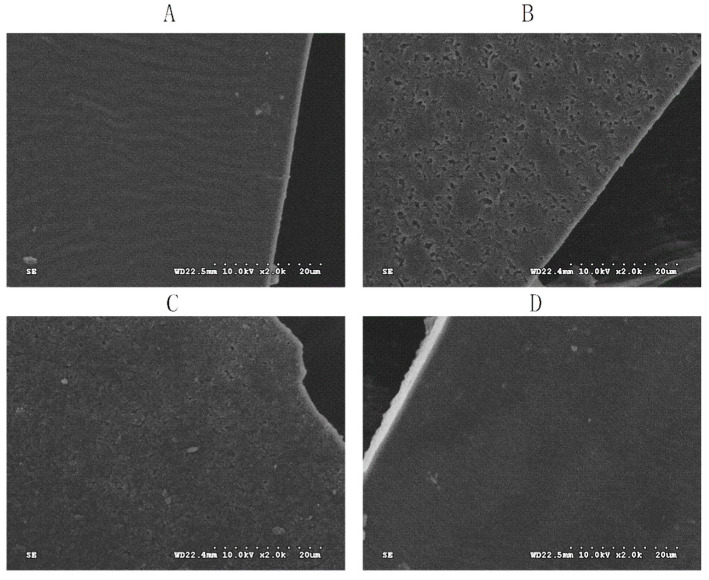
SEM of the surface (×2000) of CS (**A**), PA-g-CS-I (**B**), PA-g-CS-II (**C**), and PA-g-CS-III (**D**) films.

The surface morphology of the CS and PA-g-CS films is presented in Figure 2. The SEM showed that the CS film presented a continuous smooth surface due to the even distribution of glycerol on the entire film. However, a large number of fine cavities were observed on the PA-g-CS-I film. These cavities diminished in number with an increase in the grafting ratio, with the surface of the PA-g-CS-III film being the smoothest. Given the stronger heterogeneity of the PA-g-CS solution compared to the CS solution, the grafting of PA rendered the surface of the CS film rougher and more heterogeneous [34]. Similar phenomena were also observed in other types of phenolic acid-g-CS films [35]. The microscopic differences observed among the PA-g-CS films may be the main reason for the differences seen in their WVP, OP, and mechanical strength values.

#### 3.1.3. Light Transmission

**Figure 3:**
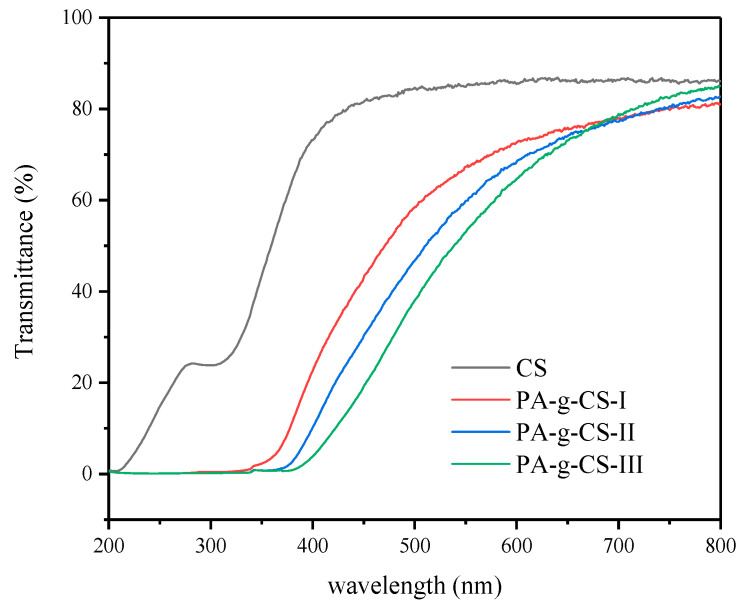
Light transmission of the CS and PA-g-CS (I-III) films.

In recent years, scholars have widely emphasized the importance of UV-protective films, since the free radicals produced from light can compromise the food quality [36,37]. As shown in Figure 3, the PA-g-CS film can shield more than 99% of UV rays, as observed in the range of 200–374 nm. The UV transmittance was inversely related to the grafting ratios between 374–400 nm. At 400 nm, PA-g-CS-I, PA-g-CS-II, and PA-g-CS-III showed the highest UV transmittance, with the values of 22.58%, 10.20%, and 3.87%, respectively, at which the transmittance of the CS film was observed to be 73.14%. Hence, our results suggested that the PA-g-CS films could effectively prevent UV light and be used to preserve the packaged food against light-related quality losses. Notably, the PA-g-CS-III film outperformed others in terms of UV-shielding capacity, owing to the formation of more C=O and C=N bonds, along with more benzene rings introduced during the grafting processes [34]. 

#### 3.1.4. Moisture Content (MC) and Water Solubility (WS)

**Figure 4:**
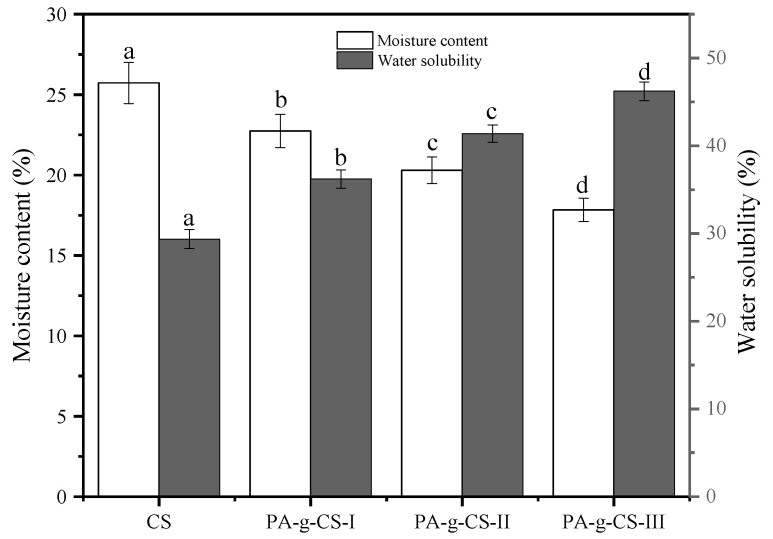
Moisture content and water solubility of the CS and PA-g-CS (I-III) films. Different letters in the same index indicate a significant difference (*p* < 0.05).

The moisture content (MC) and water solubility (WS) are the parameters that assess water sensitivity, which is one of the prominent issues with the films used for food packaging. According to Figure 4, the MC value of the CS film was found to be 25.72 ± 1.28%, and the probable cause for such a high MC value could be the powerful hydrogen bonding between the functional groups of CS and water molecules [35]. Compared to the CS film, the PA-g-CS films exhibited significantly lower MC values (*p* < 0.05), which decreased with the increase in grafting ratios. Meanwhile, the WS of the PA-g-CS films were prominently higher than for the CS film (*p* < 0.05), which increased with the increase in grafting ratio (*p* < 0.05). The possible reason behind the difference in the values of MC and WS between the CS and PA-g-CS films may be attributed to the drastic impairment of the hydrogen bonding due to the incorporation of PA. This leads to a change in the hydrophilicity of PA-g-CS. Similar findings have also been reported for the chitosan films grafted with protocatechuic acid [20], salicylic acid [38], and caffeic acid [39].

#### 3.1.5. Water Vapor Permeability (WVP) 

**Figure 5:**
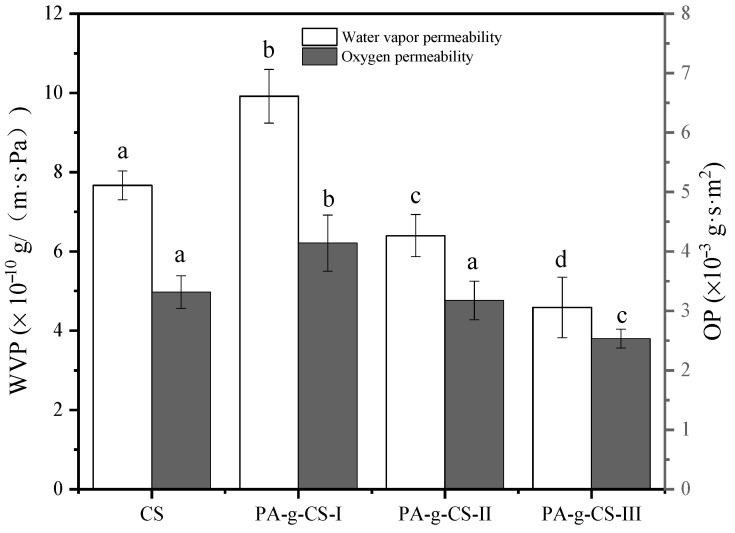
Water vapor permeability and oxygen permeability of the CS and PA-g-CS (I-III) films. Different letters in the same index indicate a significant difference (*p* < 0.05).

The WVP of the film represents the barrier property of the packaging material towards moisture. According to Figure 5, the PA-g-CS-I film exhibited significantly higher WVP than the CS film group (*p* < 0.05), whereas the WVP values in the PA-g-CS-II and PA-g-CS-III films showed a significant decrease. The WVP value of the PA-g-CS film showed a negative correlation with the grafting rate (*p* <0.05). There were several reasons behind the differences observed in the WVP values. First, as shown by SEM, different films had different gaps, leading to different WVP values, and second, the hydrophilic groups were reduced by the covalent bonding between CS and PA, causing the reduction in WVP. Besides, the inner architecture of the CS film might also be hindered by the huge benzene rings in the PA moieties [40]. In a study of the CS film grafted with protocatechuic acid by Liu et al. [20], a negative association was observed in the WVP values and the grafting ratios. According to a report by Schreiber et al. [41], the WVP value of the CS film was unaffected by gallic acid grafting. The factors that contribute to the WVP disparity among the polyphenols-grafted CS films might include the following: the molecular weight and deacetylation degree of CS, the type and dosage of the plasticizer, the process of film formation, and the type of grafted polyphenols.

#### 3.1.6. Oxygen Permeability (OP)

Oxidation is a process occurring in the presence of oxygen, which can induce alteration in food properties, including color, odor, flavor, and nutritional degradation. Accordingly, the quality and shelf-life of foods can be improved by adequate oxygen blocking measures [42]. As shown in Figure 5, the OP of the PA-g-CS-I film was found to be significantly higher than that of the other films (*p* < 0.05). With an increase in the ratio of PA grafting, the OP value of PA-g-CS tends to decrease (*p* < 0.05). The difference in the OP value was related to the surface morphology of the films.

#### 3.1.7. Tensile Strength (TS) and Elongation at Break (EB) 

**Figure 6:**
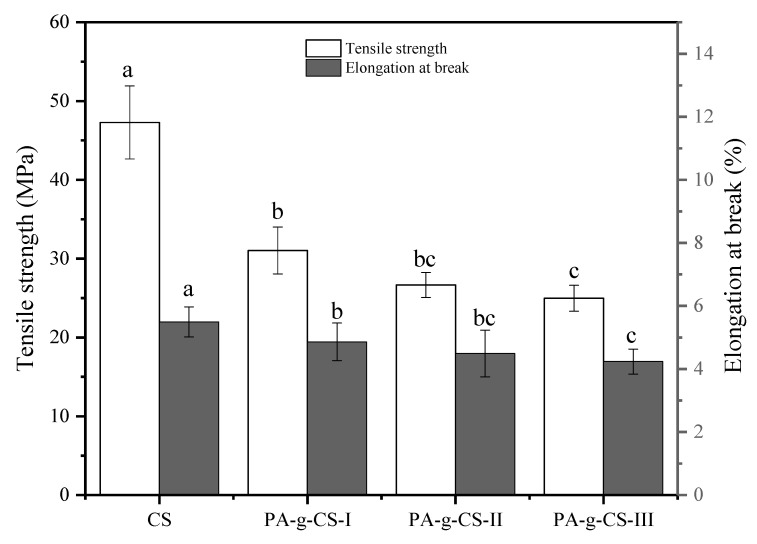
Tensile strength and elongation at break of the CS and PA-g-CS (I-III) films. Different letters in the same index indicate a significant difference (*p* < 0.05).

The mechanical behavior of a film can be represented by two major parameters, TS and EB. Here, TS refers to the endurance limit of a film that can withstand an applied tensile force, while EB reflects the stretching capability of a film [33]. TS and EB values of the CS and PA-g-CS films are presented in Figure 6. Significantly lower TS and EB values for the PA-g-CS films were observed in comparison to those for the CS film group (*p* < 0.05). In the PA-g-CS films, both TS and EB were associated negatively with the ratio of PA grafting, suggesting compromised mechanical performance after the conjugation of CS with PA. The probable reason for this may be the quantitative decrease in the functional groups of CS by PA grafting, which makes the hydrogen bonding interaction between glycerol and PA-g-CS difficult [21]. Additionally, Wang et al. [33], Aljawish et al. [35], and Rivero et al. [43] believed that the mechanical performance of the film could be affected by the MC parameter. Given the plasticizer-like action of water, a low MC could reduce the plasticity of films. The enzymatic conjugation of ferulic acid was proven to compromise the TS and EB properties of the CS films [35]. However, Zhang et al. [34] found that the TS value of the CS film was significantly elevated by the functionalization with gallic acid, while Wang observed enhanced TS and EB properties of the CS film after grafting it with caffeic acid [33]. The reason for the differences may be the types of grafts, grafting methods, and the film-forming processes.

#### 3.1.8. Antioxidant Activity of the Films

**Figure 7:**
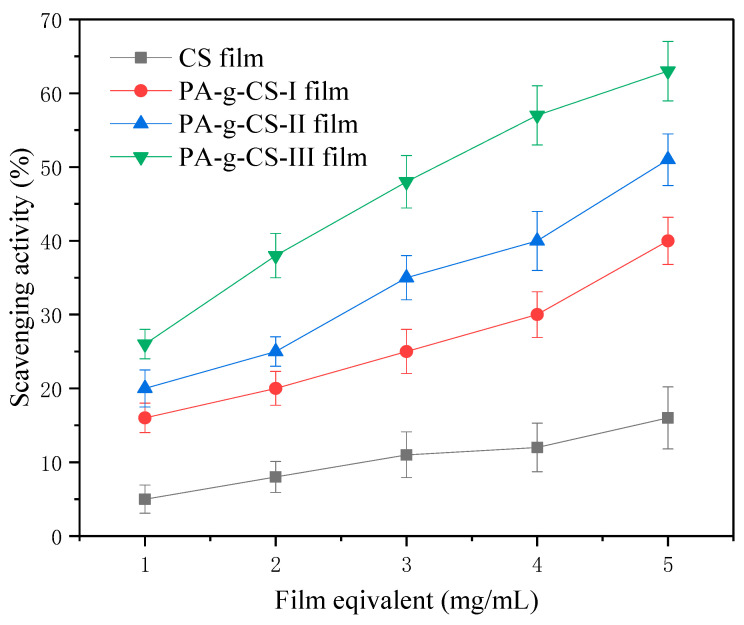
DPPH radical scavenging activity of the CS and PA-g-CS films.

The DPPH assay was employed to examine the antioxidant activity of the various studied films. The reduction in the absorbance at 517 nm was regarded as the determinant of the DPPH radical scavenging capacities of a film. As observed in Figure 7, the weakest DPPH scavenging capacity was observed in the CS film. The antioxidant activity of the CS film was attributable to the reactions of free radicals with the amino (C – 2 position) and hydroxyl groups (C – 3/C – 6 positions) [44]. The DPPH scavenging capacity of the PA-g-CS film was more potent than that of the CS film, which was positively correlated with the grafting degree. The polyphenol–chitosan conjugate was confirmed to exhibit stronger antioxidant activity [45], with PA-g-CS being easier to dissolve, making the relative concentration higher and the ability to scavenge DPPH free radicals stronger.

#### 3.1.9. Antibacterial Activity

Two common pathogens, *E. coli* (G−) and *S. aureus* (G+), were used to assess the antibacterial performance of various studied films, whose results are listed in Table 1. Compared to the CS film, a remarkably stronger (*p* < 0.05) antibacterial activity was noted in the PA-g-CS films, suggesting an enhanced antimicrobial performance of the CS film by the PA grafting. According to the extant studies, the antibacterial action can be strengthened by the synergistic interaction of PA and CS [17,34]. The high water solubility of PA-g-CS also makes it easier to handle. The CS and PA-g-CS films all exhibited stronger resistance against *S. aureus* compared to *E. coli*. The antibacterial activity against *S. aureus* of the CS and PA-g-CS films was higher than that against *E. coli.* Which may be related to the fact that the lipopolysaccharide layer on the cell membrane of Gram-negative bacteria can effectively prevent the antimicrobial substances from crossing the cell membrane, giving it certain drug resistance [38].

### 3.2. The Preservation Effect on Fresh-Cut Jackfruit

#### 3.2.1. Weight Loss and Hardness 

**Figure 8:**
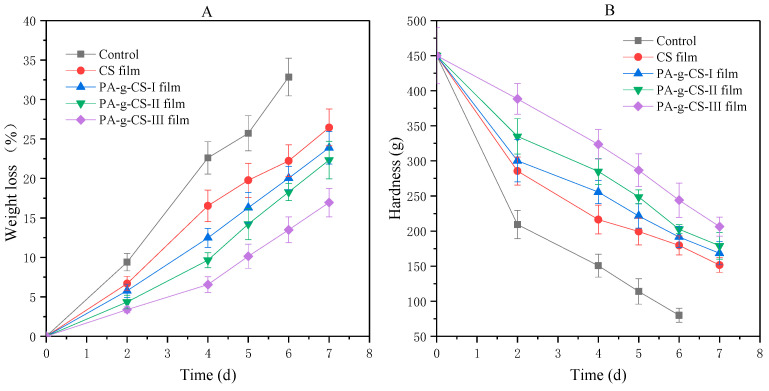
The changes in loss (**A**) and hardness (**B**) of fresh-cut jackfruit.

Factors such as water transpiration, respiration consumption, and the leakage of juice often lead to the reduction in the weight of fresh-cut fruits during storage. As shown in Figure 8A, the control group showed the greatest weight loss during storage (*p* < 0.05). Comparatively, both CS and PA-g-CS film groups exhibited lower weight reductions in the fruit. This might be attributed to a protective barrier established by the films, which could help to limit the gas exchange, while decreasing moisture evaporation and reducing the loss of organic matter. Similar delay in weight loss behavior has been demonstrated in CS-coated pummelos [31], litchis [46], and grapes [47]. 

Another serious concern in the preservation of fresh-cut fruit is rapid tissue softening, which decreases the textural quality and shortens the storage life of the products [48]. As observed in Figure 8B, the hardness of fresh-cut jackfruit continuously decreased during storage. Both CS and PA-g-CS films can significantly delay the softening of jackfruit pulp compared to the control group. (*p* < 0.05). The softening observed for the PA-g-CS-III film group was significantly slower than the other groups (*p* < 0.05). 

#### 3.2.2. The Soluble Solids (TSS) and Ascorbic Acid Content

**Figure 9:**
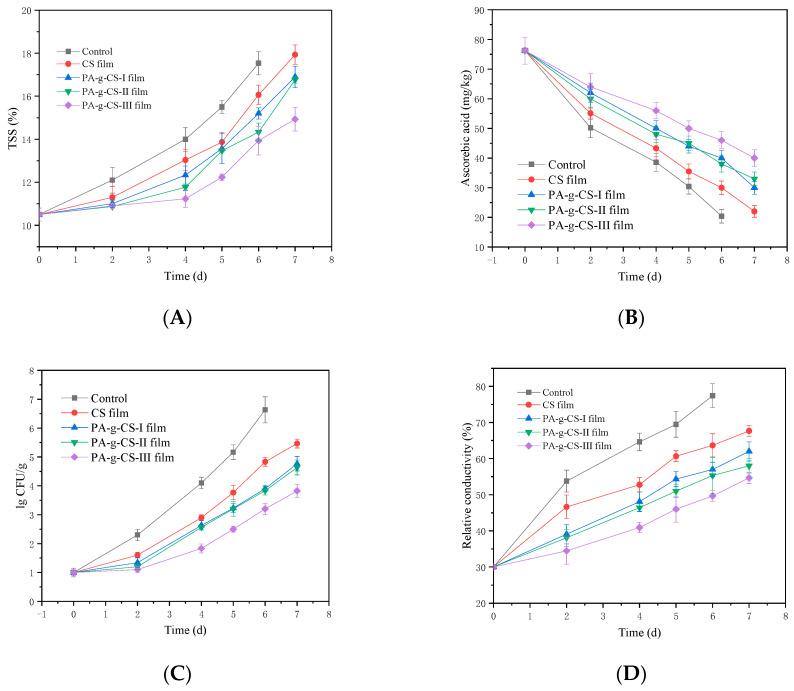

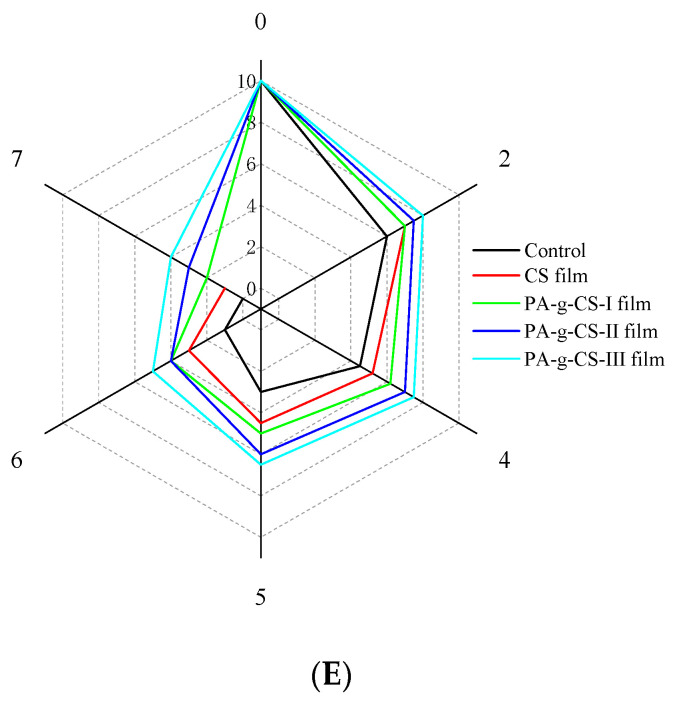
Soluble solids (TSS) (**A**), ascorbic acid content (**B**), total bacterial count (**C**), relative conductivity (**D**), and sensory evaluation (**E**) of fresh-cut jackfruit.

The TSS content is also an important indicator that reflects the fruit flavor quality. As observed in Figure 9A, the TSS of fresh-cut jackfruit showed an upward trend during storage. On the second day, the differences in the TSS value among various film groups were found to be insignificant. After the second day, the TSS of the control group increased rapidly compared to the CS and PA-g-CS film group. The slowest increase in TSS was observed in PA-g-CS-III group. The results indicate that the PA-g-CS-III film could effectively delay the ripening and senescence of jackfruit buds during storage.

Ascorbic acid content is an important nutritional quality index in fruits and vegetables [49]. The changes in ascorbic acid content of fresh-cut jackfruit during the storage period were shown in Figure 9B. Ascorbic acid content reduced with the increase in storage time. Film treatment significantly reduced (*p* < 0.05) the loss of ascorbic acid content as compared to the control during storage. The PA-g-CS film treatment also significantly reduced (*p* < 0.05) the loss of ascorbic acid content as compared to the CS film group. The ascorbic acid content for the PA-g-CS-III film treatment of jackfruit was higher in comparison to other treatments at the end of storage.

#### 3.2.3. Total Bacterial Count

Figure 9C showed the changes in the total bacterial count of fresh-cut jackfruit. During the storage period, the total bacterial count of fresh-cut jackfruit packaged in different films showed an upward trend. The total bacterial count in the control group was significantly higher than in the films group (*p* < 0.05). The total bacterial count in the CS film group was significantly higher than in the PA-g-CS films group. The total bacterial count in the PA-g-CS films group was negatively correlated with the degree of grafting. A high grafting degree of the PA-g-CS membrane could effectively reduce the colony growth in fresh-cut jackfruit.

#### 3.2.4. Membrane Relative Conductivity

The membrane intactness and permeability are often represented as the relative conductivity, which is used as a common index. A higher relative conductivity signifies severe damage to cellular membranes. As shown in Figure 9D, elevations were noted in the relative conductivity of jackfruit as the storage time was prolonged. From the second day onwards, significantly higher relative conductivity was found in the control group compared to all other groups (*p* < 0.05), while the relative conductivity of the CS film group was found to be prominently higher than the other three PA-g-CS groups (*p* < 0.05). In the PA-g-CS film groups, the grafting rate was negatively correlated with the relative conductivity, which showed that the CS grafted with PA could protect cells more effectively. Moreover, the higher the grafting rate, the better the effect.

#### 3.2.5. The Sensory Evaluation 

Fresh jackfruit flesh is golden yellow in color, full and bright in appearance, and crisp and tender in taste. Figure 9E illustrates the variations in the sensory quality of jackfruit during storage. A downward trend in sensory scores was noted in all fresh-cut jackfruits. Additionally, jackfruits covered with CS and PA-g-CS films showed better sensory properties compared to the control group. On the fourth day of storage, the sensory score dropped to 5.0 in the control group, signifying the inedibility of the corresponding fresh-cut jackfruit. On the other hand, jackfruit covered with various CS films showed improved scores of 5.2 (CS film), 6.2 (PA-g-CS-I film), 7.0 (PA-g-CS-II film), and 7.5 (PA-g-CS-III film). The highest sensory score was found in the PA-g-CS-III group, suggesting that its sensory appearance could be acceptable for marketing. 

PA-g-CS films are closely related to the oxygen permeability, water vapor transmission rate, UV blocking ability, etc. Compared to the CS film, the PA-g-CS films showed stronger antioxidant and antibacterial effects, illustrating positive associations of the conjugation of PA with CS. The films provide a relatively closed environment and a physical barrier for fresh-cut jackfruit, which protect the pulp from the external environment, and then effectively protect the fresh-cut jackfruit cells from oxidation and microbial destruction. Thus, the CS and PA-g-CS films could inhibit weight loss; maintain firmness, soluble solids, ascorbic acid content, and sensory quality; protect the cell membrane; and reduce colony count in fresh-cut jackfruit. The PA-g-CS film groups exhibited a more potent freshness-preservation effect than the CS film group, particularly the PA-g-CS-III film. 

## 4. Conclusions

The results of this work showed that the conjugated PA influenced the functional properties of the CS film and further affected its preservation of fresh-cut jackfruit. The conjugation of PA with CS enhanced water solubility, UV blocking ability, antioxidant ability, and antibacterial activities of the CS film; meanwhile, the moisture content and mechanical properties of the CS film were reduced by conjugated PA. Compared to the CS film, PA-g-CS films, particularly the PA-g-CS-III film, showed a better preservation effect on fresh-cut jackfruits in term of weight loss, hardness, soluble solids value, ascorbic acid content, total bacterial count, relative conductivity, and sensory score. The related mechanism requires future investigation. Phenolic acids (PA) showed a negative effect on the mechanical properties and a positive effect on the bioactivities of the CS film. Further research should focus on improving the mechanical property of phenolic acid-chitosan films, which will provide some idea for its commercial production. In conclusion, the conjugation of PA with CS is a more efficient way to improve the properties of CS film, and PA-g-CS conjugated films would have an extensive range of application in the food packaging industry.

## Figures and Tables

**Table 1:** Antibacterial activity of the CS and PA-g-CS films. Different letters indicate a significant difference (*p* < 0.05).

	Inhibition Zone Diameter (mm)			
	CS Film	PA-g-CS-I Film	PA-g-CS-II Film	PA-g-CS-III Film
*E. coli*	11.3 ± 0.6 ^a^	13.9 ± 0.5 ^b^	16.3 ± 0.8 ^c^	29.0 ± 1.0 ^d^
*S. aureus*	13.4 ± 0.6 ^b^	15.2 ± 0.6 ^e^	18.7 ± 0.8 ^f^	22.2 ± 1.1 ^g^

## Data Availability

The data presented in this study are available on request from the corresponding author.
